# Ritualistic Male–Male Combat of the Northern King Cobra ( *Ophiophagus hannah* ) in Thailand

**DOI:** 10.1002/ece3.71191

**Published:** 2025-03-27

**Authors:** David Roman Bontrager, Jack T. Christie, Andrew J. Pierce, Taksin Artchawakom, Surachit Waengsothorn, Max Dolton Jones

**Affiliations:** ^1^ Department of Biological Sciences Boise State University Boise Idaho USA; ^2^ Sakaerat Environmental Research Station Nakhon Ratchasima Thailand; ^3^ King Mongkut's University of Technology Thonburi Bangkok Thailand; ^4^ Population and Community Development Association Bangkok Thailand; ^5^ School of Biology Suranaree University of Technology Nakhon Ratchasima Thailand; ^6^ Department of Fish and Wildlife Conservation Virginia Tech Blacksburg Virginia USA

**Keywords:** breeding behavior, competition, Elapidae, natural history, reproduction, sexual dimorphism, snake

## Abstract

Ritualistic male–male combat is exhibited by several snake species, and is accepted as a given natural history trait for king cobras. However, there are no detailed accounts of combat behavior in king cobras in the primary literature, despite this understanding and anecdotal reporting (e.g., via social media posts). The recent taxonomic split of the king cobra species complex has increased our overall understanding of king cobras, but has narrowed the applicability of accepted knowledge across the four novel species. Here, we document three direct and indirect observations of ritualistic male–male combat in the newly revised northern king cobra ( *Ophiophagus hannah* ) in Thailand during the 2019 breeding season. We provide detailed accounts of each combat event and the implications that these observations have for our understanding of male–male combat, particularly among king cobra species and the northern king cobra specifically.

## Graphical abstract

Following the recent taxonomic split of the king cobra complex, it is important to provide new information and context to behaviors and natural history linked to each new species. We therefore provide detailed observations of ritualistic male–male combat of the newly described northern king cobra ( *Ophiophagus hannah* ). Observations were all made in Thailand during the 2019 breeding season and provide important information to further our understanding of male–male combat in snakes and the northern king cobra specifically.

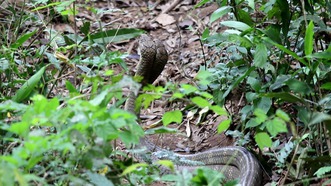

Ritualistic male–male combat in king cobras (*Ophiophagus* sp.) is a generally accepted natural history trait within much of its known distribution (Carpenter 1986; Shine 1994). Despite being repeatedly documented in non‐scientific channels, there is a lack of peer‐reviewed sources where the specifics of male–male combat in king cobras are recorded (e.g., date, time, behavior). Combat between males occurs in a wide variety of snake species and is assumed to relate to reproductive fitness as individuals compete for mating access to females (Shine 1994). While this behavior has been described in detail in some species (e.g., Malayan pit viper (Strine et al. 2015); whip snake (Baker et al. 2021); yellow‐bellied puffing snake (Araújo et al. 2024)), the distinct lack of detailed observations of king cobra combat represents a gap in our knowledge of reproductive and breeding season behaviors of the species.

Recent taxonomic revisions have identified four species of king cobras found throughout the Indian subcontinent and Southeast Asia, and here we focus on the northern king cobra ( *O. hannah* ) (Das et al. 2024; Stuart et al. 2011). Northern king cobras inhabit forests and semi‐natural areas within agricultural matrices, at times in close proximity to human populations and in highly disturbed and fragmented habitats (Jones et al. 2022; Marshall et al. 2019). Male snakes that perform ritualistic combat typically grow larger than conspecific females (Shine 1994), and male northern king cobras exhibit the same pronounced sexual size dimorphism (Marshall et al. 2018), which has also been shown in the Western Ghats king cobra (*O. kalinga*) (Shankar et al. 2013).

We describe three observations/occurrences of ritualistic male–male combat in northern king cobras in Thailand during the 2019 breeding season (approximately 10 March—05 July; Jones et al. 2022). The first direct observation occurred on Sunday, 24 March 2019, at 14:00–14:30 h at the Ban Krang campsite of Kaeng Krachan National Park, Petchaburi Province, Thailand, at (12.797976° N, 99.453610° E). Two northern king cobras were found already engaged in combat, intertwined around each other among low vegetation. The two individuals were of similar size and believed to be approximately 3.5 to 4 m in total length. The heads of both snakes were held up off the ground as they continuously intertwined the lengths of their bodies (Figure 1). Each repeatedly attempted to force the other's head downward by gaining the upper position and pushing from behind with its own head. Their main body mass shifted only slightly along the ground due to the twisting and intertwining of the bodies, covering no more than 1–2 m in total distance. They continued this intertwining and head pushing for nearly 30 min until an impending storm forced observations to stop and no further sightings of either snake were made. No biting occurred, and neither individual exhibited hooding behavior. Neither snake was visible in the combat location after the storm had passed, and no female conspecifics were observed. We only observed these individuals and therefore did not conduct cloacal probing, but based on their size and biometric records from another population of northern king cobras (Marshall et al. 2018), both were presumed to be males. The observations were made from 15 m away and the snakes appeared unaffected while observers recorded the event.

**FIGURE 1:**
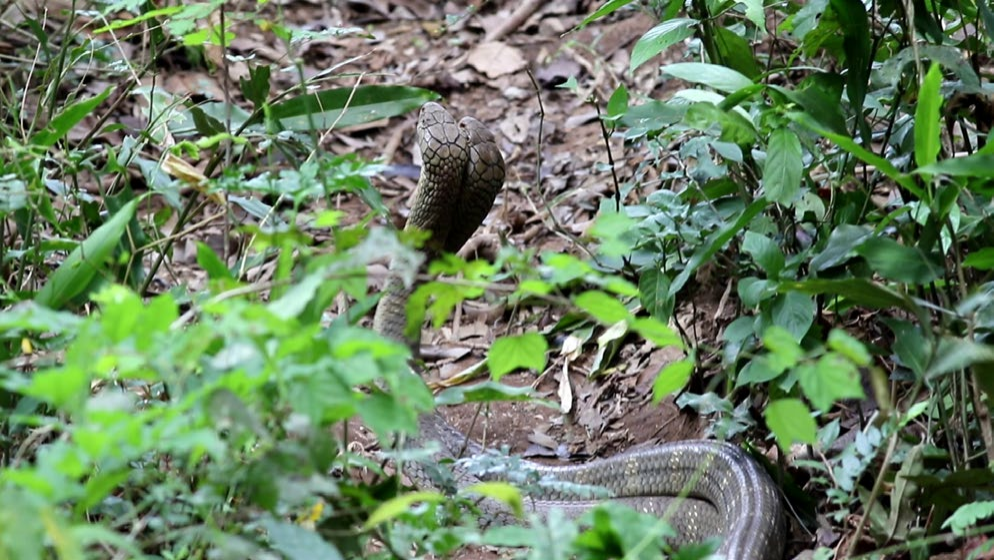
Two adult male northern king cobras ( *Ophiophagus hannah* ) engaged in ritualistic combat in Kaeng Krachan National Park, Petchaburi Province, Thailand on Sunday 24 March 2019 at 14:00 h. The heads of both individuals are raised off the ground in an attempt to push the other's head downward. Photo credit: Andrew J. Pierce.

On Saturday 30 March 2019 at 08:30 h, we discovered two separate male northern king cobras engaged in ritualistic combat. This observation occurred in the transitional zone of the Sakaerat Biosphere Reserve, Nakhon Ratchasima Province, Thailand at (14.496111° N, 101.954722° E). The site of the combat was near heterogeneous disturbed forest, plantation forest, agricultural fields, paved and dirt roads, and other human‐made structures (bridge, building, and reservoir). We approached an open clearing 20 m from a paved road with sparse low vegetation surrounding a large rock complex. In the open area, two large male northern king cobras (adult male 006 [AM006]: 3.5 m [biometric data collected on 18 October 2018]; and adult male 007 [AM007]: 3.8 m [biometric data collected on 30 March 2019]; sex determined via cloacal probing) were found with their bodies fully elongated and intertwined (Figure 2). The anterior portions of both snakes were elevated off the ground, and each was attempting to push the head of the other downwards from behind. They slowly moved over the course of the interaction, covering 10–15 m in total distance in various directions. Their bodies remained entangled during the entire combat episode. No biting occurred, and neither individual exhibited hooding behavior. At 08:44 h, the two snakes separated, and the apparent loser of the interaction retreated away from the site toward a nearby rock complex, and the suspected victor—which was the larger individual—remained at the combat site. No female conspecifics were observed. Observations were made from 30 m away, and our presence did not appear to affect their behavior.

**FIGURE 2:**
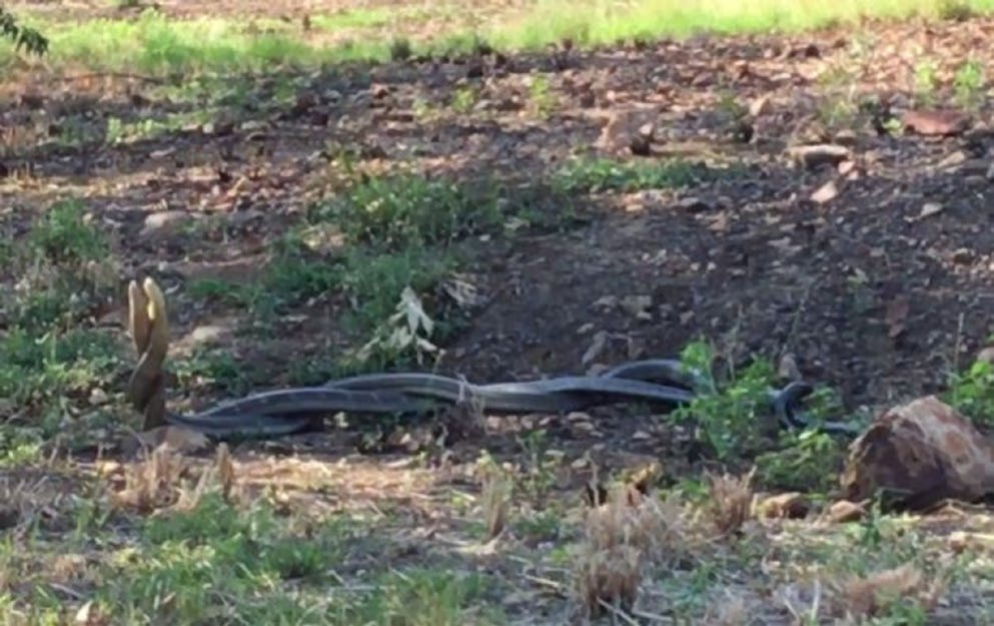
Two adult male northern king cobras ( *Ophiophagus hannah* ) engaged in ritualistic combat in the transitional zone of the Sakaerat Biosphere Reserve, Nakhon Ratchasima Province, Thailand on Saturday 30 March 2019 at 08:30 h. While their heads are raised off the ground to initiate a downward head push, the entire lengths of their bodies are entangled in plaiting combat. Photo credit: David R. Bontrager.

The third case of male–male combat occurred on Saturday 6 April 2019 in an agricultural area within the transitional zone of Sakaerat Biosphere Reserve. This combat event was not observed directly by our team, but using radio telemetry data (adult male 054 [AM054]: 3.2 m [biometric data collected on 20 November 2018]; and adult male 059 [AM059]: 3.6 m [biometric data collected on 28 March 2019]; sex determined via cloacal probing) and a description from a local community member, we are highly confident that a combat occurred. We subset our tracking data to a 2‐week period before and after the suspected combat event. Since AM059 was only added to the project a few days before the event, the subset data for this male only spans 3 days before. We followed methodology highlighted in Jones et al. (2022) and ran dynamic Brownian Bridge Movement Models (dBBMM) on each movement subset using the move v.4.2.4 package (Kranstauber et al. 2023) in program R v.4.4.0 (R Core Team 2024) through Rstudio v.2024.4.1.748 (Posit Team 2024). We extracted the 90%, 95%, and 99% contours to produce a movement pathway for each snake, and overlapped all the contours to visualize where the pathways intersected (Figure 3). Using the telemetry fixes, we can see that AM054 moved to a new location on 5 April 2019, and AM059 began moving north. By the end of the next day, AM059 had displaced AM054 from the new location, and AM054 moved away from the location where their fixes overlapped. By 12 April 2019, AM054 had shown no further movement, and AM059 had begun to move away from the area (Figure 3). This evidence suggests that they had interacted with each other at some point during the exchange of the shelter site. Additionally, during the process of tracking these individuals on 6th April, a local community member nearby communicated with our telemetry team that they had seen two snakes engaged in combat, twisting their arms together vigorously to mimic the intertwining behavior of the snakes. Because AM054 was displaced at the shelter site by AM059 (Figure 3), we postulate that AM054 lost the combat, while AM059, the larger of the pair, remained.

**FIGURE 3:**
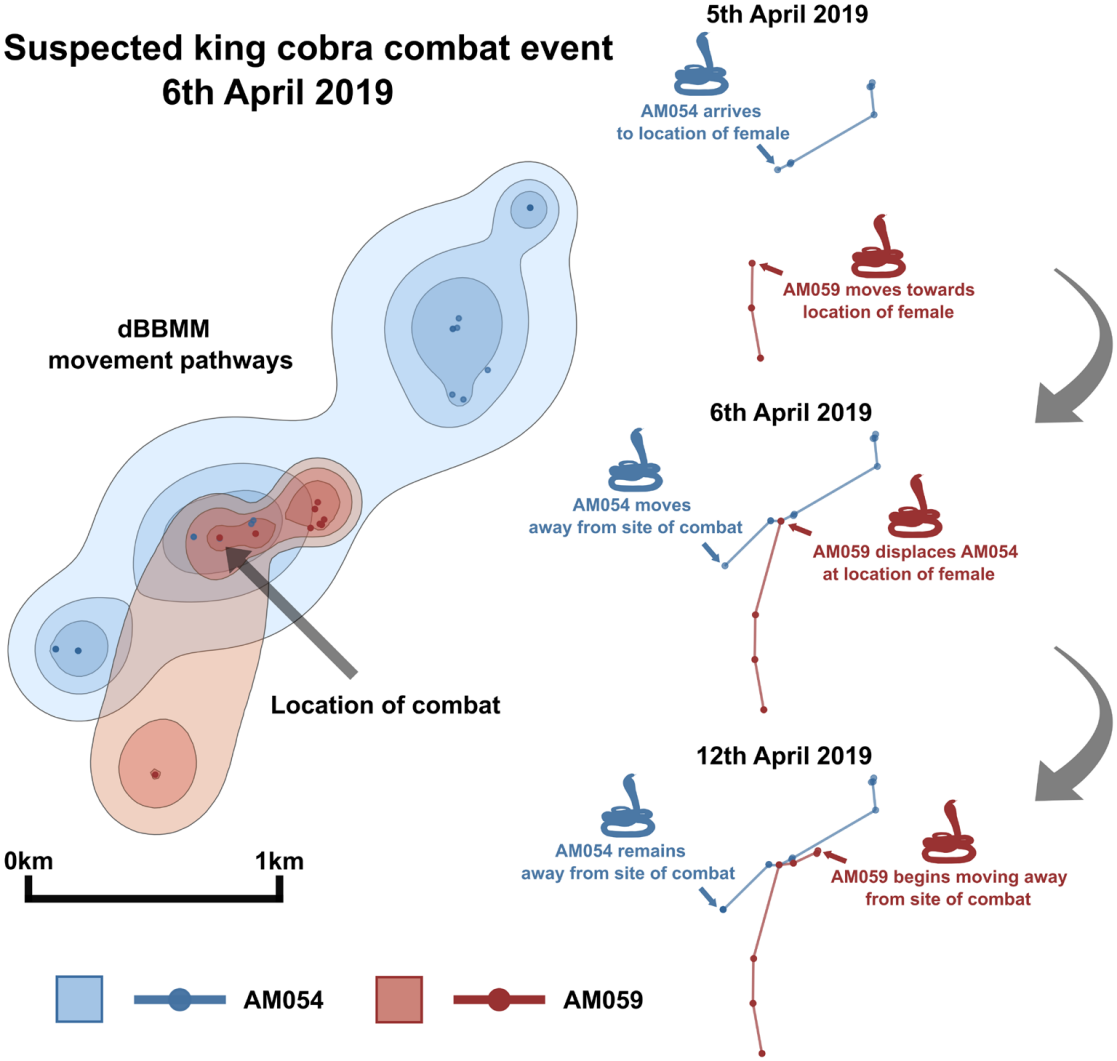
Evidence for a suspected northern king cobra ( *Ophiophagus hannah* ) combat event on 6 April 2019. Dynamic Brownian bridge movement model (dBBMM) for movement pathways display 90%, 95%, and 99% dBBMM contours with decreasing opacity. Scale bar indicates scale of dBBMM contours and associated points. Fixes and trajectory lines on right of figure are not to scale. Points, contours, and trajectory lines for AM054 and AM059 are depicted using blue and red, respectively.

Our understanding of the recently described northern king cobra has expanded significantly in recent years, including new insights into their diet (Jones et al. 2020), space use and resource selection (Marshall et al. 2019, 2020), and anthropogenic risks and threats (Jones et al. 2022; Marshall et al. 2018). These observations of ritualistic male–male combat in northern king cobras continue to improve our understanding of the species, particularly as more is learned about the king cobra species complex (Das et al. 2024). The similarities between the different combat observations are evidence that combat is a breeding season behavior among male northern king cobras in various parts of its range in Thailand. This behavior has been documented in other snake species (Senter 2022; Shine 1994), and is known to occur in *O. kalinga* in India via multiple lines of evidence outside of the primary literature (e.g., social media, blogs, videos). Our observations provide insight into the natural history of the species and inform our understanding of selective pressures and intraspecific competition.

The specific behaviors during these combat episodes have been described previously in other species by Senter (2022), and are known to occur in other Elapids including black mambas ( *Dendroaspis polylepis* ), a close relative of the king cobra species complex (Pyron et al. 2013). All individuals that we observed exhibited the Type 2 Head Raise and the Downward Push. The Downward Push, described as “both snakes have anterior ends elevated, often coiled around each other, and each attempts to push the other toward the ground” (Senter 2022), appeared to be the focus of the combating snakes. Forcing the head of the opponent downward could largely determine the victor of the combat, with size, strength, and stamina contributing to the success of pushing the other's head down.

The lack of biting between the northern king cobras in our observations is highly notable and suggests that combat in this species may not typically result in fatalities of conspecific males. While biting does occur during combat in some snake species, it is more common in non‐venomous species (Shine 1994). Captive studies on another Elapid, the tiger snake ( *Notechis ater* ), found biting occurred in male–male combats when food was present, thus identifying a distinction between sexual and food‐related combats in the species (Firmage and Shine 1996). As its generic epithet suggests (*Ophiophagus* meaning “snake eating”), the diet of northern king cobras consists largely of other snakes, and they are often considered snake specialists, though monitor lizards have been shown to make up a significant portion of their diet as well (Jones et al. 2020; Smith et al. 2021). Importantly, during our long‐term telemetry project, we recorded cannibalistic behavior outside of the breeding season, where a large radiotracked male ate a much smaller radiotracked adult female. Additionally, cannibalism is known to occur in *O. kalinga* (Shankar and Whitaker 2013). This suggests that during the breeding season, combat appears to be a distinct behavior from hunting or predatory behaviors and would therefore not likely result in either snake being killed or consumed. The combatting pairs in our observations were of similar size; however, the fact that our observations occurred with similarly sized individuals may be due to combat events of similarly sized individuals lasting longer than those between individuals differing greatly in size. An evenly matched combat may take longer to conclude, thus giving observers more time to actually find and record these events. We could therefore be missing many observations of male–male combat in northern king cobras, as well as in the other *Ophiophagus* species, particularly uneven matchups that have very short combat durations between individuals that differ greatly in size. Additionally, it is notable that neither case of observed combat involved the distinctive king cobra hooding behavior, which is often used as a defensive display (Panagides et al. 2017); showing further that context is important and combat is distinctive of other behaviors. Though the heads of all the individuals were raised off the ground in a similar manner to a typical hooding display, the nuchal regions of all snakes involved were not splayed, suggesting that combat also differs from a defensive display.

Combat between male northern king cobras, as we observed, is likely primarily related to gaining access to females for mating. The presumed winners of the second and third observed combats stayed at the sites of combat while the suspected losers left the area. This is likely to rest and recover following an energy‐intensive combat before seeking out a female that, though we never observed, was suspected to be nearby. These presumed winners were slightly larger than their opponents (confirmed using biometric data collection), suggesting that larger and heavier individuals have greater success in pushing their opponents downward and therefore more opportunities for subsequent mating with females. Male–male combat selects for larger males than would occur without combat, resulting in sexual size dimorphism over time, assuming similar selective pressures for larger size do not occur in females (Shine 1994). Though sexual size dimorphism can occur in species without male–male combat, these species generally have larger females than males (Shine 1994). Species with larger males and no documentation of combat may suggest other factors also influence this trait; however, it is possible that many of those species do exhibit male–male combat but it has not yet been observed (Senter 2022; Shine 1978). Our observations support that the apparent sexual size dimorphism in king cobras is in part due to ritualistic male–male combat, with victory leading to more mating opportunities.

Competition for resources other than mates, including food, may also lead to ritualistic combat that is behaviorally similar to combat over mating opportunities (Farrell et al. 2024), and could have been the impetus for any one of the described combat events. However, while we did not observe female northern king cobras near any of the combat events, no potential prey items were observed either, and all three observations occurred in a short time period within our previously delineated breeding season (Jones et al. 2022). We have also observed male northern king cobras sheltering in the same shelter sites outside of the breeding season, which suggests that they may share certain resources rather than engage in combat over them during the non‐breeding season. The cannibalism observed in this population also occurred outside of the breeding season, while our breeding season combat events appear to be distinct from cannibalistic or feeding behaviors. The timing of our observations, combined with the lack of cannibalistic behavior or even biting, which has been linked to combats over food in other species (Firmage and Shine 1996), suggests that the observed combats were likely over mate acquisition.

The similarities between the combats we observed suggest these behaviors would be common in combats across the northern king cobra's range, and potentially among the four species of king cobras. More observations are needed to confirm this, but the presence of plaiting, downward push, and head raises, and the absence of biting and hooding all suggest that king cobra combat would generally follow these behavioral patterns. However, further research and new observations will illuminate whether this behavior occurs in all four king cobra species and if there are similarities or differences in specific combat behaviors among species. With more observations of ritualistic male–male combat, we can gain a better understanding of the natural history of these species, and further our knowledge of factors affecting their reproductive success.

## Author Contributions

**David Roman Bontrager:** conceptualization (equal), data curation (equal), investigation (equal), methodology (equal), visualization (equal), writing – original draft (lead), writing – review and editing (equal). **Jack T. Christie:** conceptualization (equal), investigation (equal), methodology (equal), writing – original draft (equal), writing – review and editing (equal). **Andrew J. Pierce:** data curation (equal), investigation (equal), resources (equal), visualization (equal), writing – review and editing (equal). **Taksin Artchawakom:** project administration (equal), resources (equal), writing – review and editing (equal). **Surachit Waengsothorn:** project administration (equal), resources (equal), writing – review and editing (equal). **Max Dolton Jones:** conceptualization (equal), data curation (lead), formal analysis (lead), funding acquisition (lead), investigation (equal), methodology (equal), project administration (equal), software (equal), supervision (lead), validation (equal), visualization (lead), writing – review and editing (equal).

## Conflicts of Interest

The authors declare no conflicts of interest.

## Data Availability

Video evidence for Observation 1 and Observation 2 can be found at the Zenodo repository: https://doi.org/10.5281/zenodo.14927536. Data for the third indirect observation can be found at: https://doi.org/10.5281/zenodo.5148436.

## References

[ece371191-bib-0001] Araújo, G. , R. Ramalho , and S. Almeida‐Santos . 2024. “First Record of Male‐Male Combat in the Yellow‐Bellied Puffing Snake *Spilotes sulphureus* (Wagler, 1824).” Amphibia‐Reptilia 45, no. 4: 485–490. 10.1163/15685381-bja10194.

[ece371191-bib-0002] Baker, M. A. A. , M. Al‐Saraireh , Z. S. Amr , and P. J. Senter . 2021. “Male‐Male Combat in the Large Whip Snake, *Dolichophis jugularis* (Serpentes: Colubridae).” Herpetology Notes 14: 735–744.

[ece371191-bib-0003] Carpenter, C. C. 1986. “An Inventory of Combat Rituals in Snakes.” Smithsonian Herpetological Information Service 69, no. 69: 1–18. 10.5479/si.23317515.69.1.

[ece371191-bib-0004] Das, I. , P. G. Shankar , P. Swamy , et al. 2024. “Taxonomic Revision of the King Cobra *Ophiophagus hannah* (Cantor, 1836) Species Complex (Reptilia: Serpentes: Elapidae), with the Description of Two New Species.” European Journal of Taxonomy 961: 1–51. 10.5852/ejt.2024.961.2681.

[ece371191-bib-0005] Farrell, T. M. , H. C. Gull , F. S. Boyce , and S. C. Richter . 2024. “Ritualized Male–Male Combat Resulting From Intraspecific Food Competition in Three Agkistrodon Species.” Journal of Ethology 42, no. 2: 83–88. 10.1007/s10164-024-00806-8.

[ece371191-bib-0006] Firmage, M. , and R. Shine . 1996. “Battles for Mates and Food: Intraspecific Combat in Island Tigersnakes ( *Notechis ater* ) From Southern Australia.” Amphibia‐Reptilia 17, no. 1: 55–65. 10.1163/156853896X00298.

[ece371191-bib-0007] Jones, M. D. , M. S. Crane , I. M. S. Silva , et al. 2020. “Supposed Snake Specialist Consumes Monitor Lizards: Diet and Trophic Implications of King Cobra Feeding Ecology.” Ecology 101, no. 10: e03085. 10.1002/ecy.3085. 32320484

[ece371191-bib-0008] Jones, M. D. , B. M. Marshall , S. N. Smith , et al. 2022. “How Do King Cobras Move Across a Major Highway? Unintentional Wildlife Crossing Structures May Facilitate Movement.” Ecology and Evolution 12, no. 3: e8691. 10.1002/ece3.8691. 35342558 PMC8928851

[ece371191-bib-0009] Kranstauber, B. , M. Smolla , and A. K. Scharf . 2023. “move: Visualizing and Analyzing Animal Track Data.” R package version 4.2.4. https://CRAN.R‐project.org/package=move.

[ece371191-bib-0010] Marshall, B. M. , M. Crane , I. Silva , et al. 2020. “No Room to Roam: King Cobras Reduce Movement in Agriculture.” Movement Ecology 8, no. 1: 33. 10.1186/s40462-020-00219-5. 32774861 PMC7397683

[ece371191-bib-0011] Marshall, B. M. , C. T. Strine , M. D. Jones , et al. 2018. “Hits Close to Home: Repeated Persecution of King Cobras (*Ophiophagus hannah*) in Northeastern Thailand.” Tropical Conservation Science 11: 1940082918818401. 10.1177/1940082918818401.

[ece371191-bib-0012] Marshall, B. M. , C. T. Strine , M. D. Jones , et al. 2019. “Space Fit for a King: Spatial Ecology of King Cobras (*Ophiophagus hannah*) in Sakaerat Biosphere Reserve, Northeastern Thailand.” Amphibia‐Reptilia 40, no. 2: 163–178. 10.1163/15685381-18000008.

[ece371191-bib-0013] Panagides, N. , T. Jackson , M. Ikonomopoulou , et al. 2017. “How the Cobra Got Its Flesh‐Eating Venom: Cytotoxicity as a Defensive Innovation and Its co‐Evolution With Hooding, Aposematic Marking, and Spitting.” Toxins 9, no. 3: 103. 10.3390/toxins9030103. 28335411 PMC5371858

[ece371191-bib-0014] Posit Team . 2024. “RStudio: Integrated Development Environment for R.” Posit Software, PBC, Boston, MA. http://www.posit.co/.

[ece371191-bib-0015] Pyron, R. , F. T. Burbrink , and J. J. Wiens . 2013. “A Phylogeny and Revised Classification of Squamata, Including 4161 Species of Lizards and Snakes.” BMC Evolutionary Biology 13, no. 1: 93. 10.1186/1471-2148-13-93. 23627680 PMC3682911

[ece371191-bib-0016] R Core Team . 2024. “R: A Language and Environment for Statistical Computing.” R Foundation for Statistical Computing, Vienna, Austria. https://www.R‐project.org/.

[ece371191-bib-0017] Senter, P. J. 2022. “Phylogeny of Courtship and Male‐Male Combat Behavior in Snakes: An Updated Analysis.” Current Herpetology 41, no. 1: 35–81. 10.5358/hsj.41.35.

[ece371191-bib-0018] Shankar, P. G. , S. R. Ganesh , R. Whitaker , and P. Prashanth . 2013. “King Cobra *Ophiophagus hannah* (Cantor, 1836) Encounters in Human‐Modified Rainforests of the Western Ghats, India.” Hamadryad 36, no. 2: 62–68.

[ece371191-bib-0019] Shankar, P. G. , and R. Whitaker . 2013. “Cannibalism in Wild and Captive King Cobras *Ophiophagus hannah* (Cantor, 1836).” Hamadryad 36, no. 2: 87–90.

[ece371191-bib-0020] Shine, R. 1978. “Sexual Size Dimorphism and Male Combat in Snakes.” Oecologia 33, no. 3: 269–277. 10.1007/BF00348113. 28309592

[ece371191-bib-0021] Shine, R. 1994. “Sexual Size Dimorphism in Snakes Revisited.” Copeia 1994, no. 2: 326. 10.2307/1446982.

[ece371191-bib-0022] Smith, S. N. , M. D. Jones , B. M. Marshall , S. Waengsothorn , G. A. Gale , and C. T. Strine . 2021. “Native Burmese Pythons Exhibit Site Fidelity and Preference for Aquatic Habitats in an Agricultural Mosaic.” Scientific Reports 11, no. 1: 7014. 10.1038/s41598-021-86640-1. 33782524 PMC8007826

[ece371191-bib-0023] Strine, C. T. , C. Barnes , I. Silva , B. Nadolski , J. G. Hill , and P. Suwanwaree . 2015. “First Record of Male Combat in a Wild Malayan Pit Viper.” Asian Herpetological Research 6, no. 3: 237–239.

[ece371191-bib-0024] Stuart, B. , G. Wogan , L. Grismer , et al. 2011. “IUCN Red List of Threatened Species: *Ophiophagus hannah*. IUCN Red List of Threatened Species.” https://www.iucnredlist.org/en.

